# *Staphylococcus brunensis* sp. nov. isolated from human clinical specimens with a staphylococcal cassette chromosome-related genomic island outside of the *rlmH* gene bearing the *ccrDE* recombinase gene complex

**DOI:** 10.1128/spectrum.01342-23

**Published:** 2023-09-15

**Authors:** Vojtěch Kovařovic, Adéla Finstrlová, Ivo Sedláček, Petr Petráš, Pavel Švec, Ivana Mašlaňová, Meina Neumann-Schaal, Ondrej Šedo, Tibor Botka, Eva Staňková, Jiří Doškař, Roman Pantůček

**Affiliations:** 1 Department of Experimental Biology, Division of Genetics and Molecular Biology, Faculty of Science, Masaryk University, Brno, Czech Republic; 2 Department of Experimental Biology, Czech Collection of Microorganisms, Faculty of Science, Masaryk University, Brno, Czech Republic; 3 Reference Laboratory for Staphylococci, National Institute of Public Health, Praha, Czech Republic; 4 Leibniz Institute DSMZ-German Collection of Microorganisms and Cell Cultures, Braunschweig, Germany; 5 Central European Institute of Technology, Masaryk University, Brno, Czech Republic; Michigan State University, East Lansing, Michigan, USA

**Keywords:** coagulase-negative staphylococci, phylogenetic analysis, comparative genomics, mobile genetic elements, genomic islands, cassette chromosome recombinase, polyphasic taxonomy, gram-positive pathogens

## Abstract

Novel species of coagulase-negative staphylococci, which could serve as reservoirs of virulence and antimicrobial resistance factors for opportunistic pathogens from the genus *Staphylococcus*, are recognized in human and animal specimens due to advances in diagnostic techniques. Here, we used whole-genome sequencing, extensive biotyping, MALDI-TOF mass spectrometry, and chemotaxonomy to characterize five coagulase-negative strains from the *Staphylococcus haemolyticus* phylogenetic clade obtained from human ear swabs, wounds, and bile. Based on the results of polyphasic taxonomy, we propose the species *Staphylococcus brunensis* sp. nov. (type strain NRL/St 16/872^T^ = CCM 9024^T^ = LMG 31872^T^ = DSM 111349^T^). The genomic analysis revealed numerous variable genomic elements, including staphylococcal cassette chromosome (SCC), prophages, plasmids, and a unique 18.8 kb-long genomic island SbCI*_ccrDE_* integrated into the ribosomal protein L7 serine acetyltransferase gene *rimL*. SbCI*_ccrDE_* has a cassette chromosome recombinase (*ccr*) gene complex with a typical structure found in SCCs. Based on nucleotide and amino acid identity to other known *ccr* genes and the distinct integration site that differs from the canonical methyltransferase gene *rlmH* exploited by SCCs, we classified the *ccr* genes as novel variants, *ccrDE*. The comparative genomic analysis of SbCI*_ccrDE_* with related islands shows that they can accumulate virulence and antimicrobial resistance factors creating novel resistance elements, which reflects the evolution of SCC. The spread of these resistance islands into established pathogens such as *Staphylococcus aureus* would pose a great threat to the healthcare system.

**IMPORTANCE:**

The coagulase-negative staphylococci are important opportunistic human pathogens, which cause bloodstream and foreign body infections, mainly in immunocompromised patients. The mobile elements, primarily the staphylococcal cassette chromosome *mec*, which confers resistance to methicillin, are the key to the successful dissemination of staphylococci into healthcare and community settings. Here, we present a novel species of the *Staphylococcus* genus isolated from human clinical material. The detailed analysis of its genome revealed a previously undescribed genomic island, which is closely related to the staphylococcal cassette chromosome and has the potential to accumulate and spread virulence and resistance determinants. The island harbors a set of conserved genes required for its mobilization, which we recognized as novel cassette chromosome recombinase genes *ccrDE*. Similar islands were revealed not only in the genomes of coagulase-negative staphylococci but also in *S. aureus*. The comparative genomic study contributes substantially to the understanding of the evolution and pathogenesis of staphylococci.

## INTRODUCTION

Staphylococci, mainly coagulase-positive *Staphylococcus aureus*, are the leading cause of a broad spectrum of diseases in humans and animals. Over the last three decades, coagulase-negative staphylococcal species (CoNS), with the most significant being *Staphylococcus epidermidis*, *Staphylococcus haemolyticus,* and *Staphylococcus hominis*, have been recognized as opportunistic pathogens, especially in immunocompromised patients. CoNS are a frequent cause of nosocomial infections related to catheters or medical devices aided by their ability to form a biofilm (1). Additionally, the gene pool of substrate utilization pathways and resistance determinants enables CoNS to occupy various niches, providing favorable conditions for the emergence of multidrug-resistant CoNS and their subsequent spread in healthcare environments (2).

The adaptation of *S. haemolyticus* strains to diverse environments is facilitated by frequent recombination among numerous insertion sequences (ISSha*1*) (3). Therefore, the standard microbial and molecular diagnostic tools have limited discriminatory power to reliably distinguish species closely related to *S. haemolyticus* (4). Only recently, molecular diagnostics approaches, mainly in-depth whole-genome characterization, have assigned atypical *S. haemolyticus* strains isolated from clinical specimens into the new species *Staphylococcus borealis* (5) and *Staphylococcus taiwanensis* (6). The core genome phylogeny also led to the reclassification of the *S. petrasii* phylogenetic complex (7, 8), which now consists of three species*—S. petrasii*, *S. croceilyticus*, and *S. pragensis* (9). All these species were isolated from various human biological samples, mainly from wounds, eye and ear infections, urinary infections, and blood samples (10).

The versatility of CoNS is associated with a significant reservoir of mobile genetic elements (MGEs). Notably, the methicillin resistance encoded by the staphylococcal chromosomal cassette (SCC) *mec* (SCC*mec*) significantly complicates healthcare and increases the need to use second-line antimicrobial drugs to treat staphylococcal infections (11). Apart from *mec* genes responsible for methicillin resistance, SCC*mec* carries numerous genes for virulence, such as phenol-soluble modulins (PSM-*mec*), plasmin, or heavy-metal and other resistance genes contributing to the survival of these strains in an environment (12 – 14). It is suspected that the SCC*mec* originates from CoNS species (15), but the natural mechanism of SCC transmission is still unknown. It can be transferred intra- and even interspecies by transduction (16), conjugation (17), or natural competence (18). The transfer of SCC*mec* is mediated by *ccr* chromosome cassette genes of the serine recombinase family. Three phylogenetically distinct *ccr* genes, namely *ccrA*, *ccrB*, and *ccrC,* have been delineated with nucleotide identities below 50%. These recombinases recognize a specific *att* site in the bacterial *rlmH* gene for ribosomal RNA large-subunit methyltransferase H (19). The recombinases CcrA and CcrB function together as a heterotetramer in the specific excision of the SCC (20), whereas the CcrC recombinase enables mobilization of the element without another recombinase (21).

The recent increase in the association of CoNS with nosocomial infections and improvements in diagnostic approaches make it possible to recognize other often overlooked species of this group related to human diseases. Investigation of these CoNS as a pool of genes for antimicrobial resistance and virulence can reveal the molecular mechanisms that lead to these opportunistic pathogens’ evolution, adaptation, and success. This article reports the polyphasic characterization of five *Staphylococcus* sp. isolates from human clinical material to delineate a novel species. Whole-genome sequence analyses of the strains revealed a remarkable non-SCC genomic island harboring *ccrDE* cassette chromosome recombinase types.

## RESULTS

### Phylogenetic relationship of the strains

**Fig 1:**
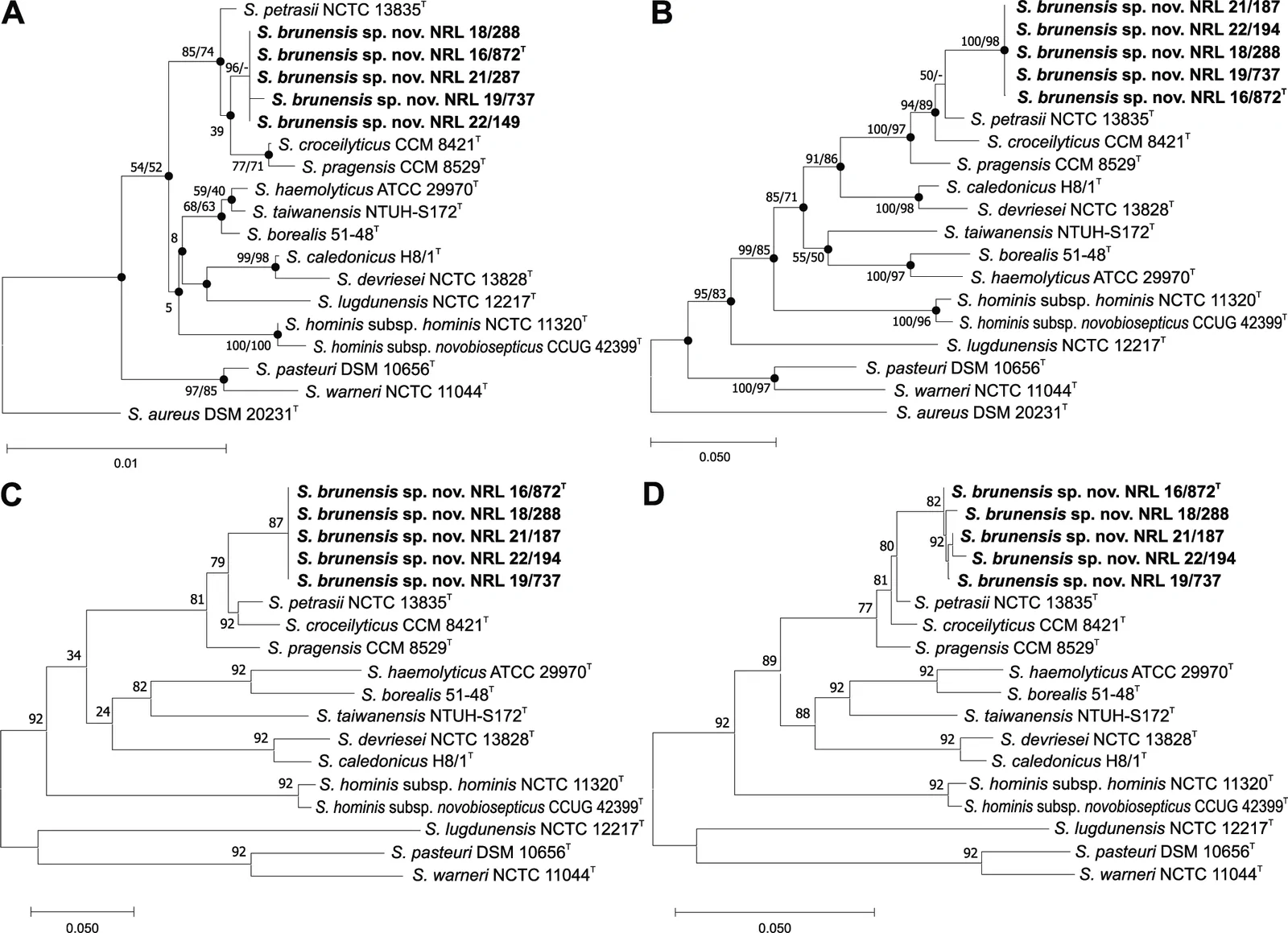
Evolutionary analyses of *Staphylococcus brunensis* sp. nov. and its closest relatives. GenBank accession numbers of used sequences are listed in Table S1. (**A**) Evolutionary history inferred using neighbor-joining (NJ) method based on complete 16S rRNA gene sequences extracted from whole-genome assemblies. Filled circles indicate that the corresponding nodes were also identified from analysis by the maximum likelihood (ML) method. The percentage of replicate trees above 50% in which the associated taxa clustered together in the bootstrap test (500 replicates) are shown next to the branches for the NJ and ML methods. *S. aureus* DSM 20231^T^ was used as an outgroup. The evolutionary distances are in the units of the number of base substitutions per site. All ambiguous positions were removed for each sequence pair. There were a total of 1,552 positions in the final data set. (**B**) Unrooted ML tree based on multilocus sequence analysis of concatenated nucleotide sequences from six loci—*rpoB*, *hsp60*, *dnaJ*, *tufA*, *gap,* and *sodA—*were extracted from whole-genome assemblies. Filled circles indicate that the corresponding nodes were also identified from analysis by the NJ method. There were a total of 3,952 positions in the final data set. Bootstrap probability values (percentages of 500 tree replications) greater than 50% are shown at branch points. The evolutionary distances are given as the number of substitutions per site. (**C**) Nucleotide sequence-based and (**D**) protein sequence-based phylogenetic tree of the concatenated alignment of 92 core genes constructed using up-to-date bacterial core gene (UBCG) set. The ML tree was inferred using RAxML software and 100 replicates. The threshold for the gene support index was set to 94% for the nucleotide and 80% for the protein-based tree. Gene support indices are given at branching points (the maximal possible value is 92). The bar indicates the number of substitutions per position.

Five unidentified *Staphylococcus* sp. strains were collected from various human clinical specimens from both mixed culture and monoculture between 2016 and 2022 (Table 1) and transferred to the National Reference Laboratory for Staphylococci (National Institute of Public Health, Prague) for long-term storage and further study. The phylogenetic analysis of complete 16S rRNA gene sequences consistently placed the five isolates in *S. haemolyticus* cluster group defined previously (22). The closest relatives were *Staphylococcus petrasii*, *Staphylococcus croceilyticus*, and *Staphylococcus pragensis,* with 16S rRNA gene sequence similarities ranging from 99.80 to 99.59%, while other species were below 99.4% similarity. The topology of the neighbor-joining (NJ) phylogenetic tree constructed with 16S rRNA gene sequences was similar to that of the maximum likelihood (ML) tree (Fig. 1A). Because the 16S rRNA analysis has limited discriminatory power in the genus *Staphylococcus* (5, 6, 23), the phylogenetic position of the new isolates was also assessed using the concatenated multilocus sequence data of six routinely used conserved housekeeping genes: *rpoB*, *hsp60*, *dnaJ*, *tufA*, *gap*, and *sodA* for discrimination of staphylococcal species (Fig. 1B). The ML phylogenetic tree for the housekeeping genes had a very similar topology to that of the 16S rRNA gene tree and to the additional phylogenetic trees constructed using the up-to-date bacterial core gene (UBCG) at the nucleotide and protein level (Fig. 1C and D).

**TABLE 1:** Origin of the strains of *Staphylococcus brunensis* sp. nov. characterized in this study

Strain	Date of isolation	Locality	Specimen	Sex	Age	Diagnosis	Other microflora
NRL/St^*a*^ 16/872^T^ = CCM^*b*^ 9024^T^ = LMG^*c*^ 31,872^T^ = DSM^*d*^ 111349^T^	Oct 2016	Karlovy Vary	Swab of ear	M	3 y	Acute otitis externa	Monoculture
NRL/St^*a*^ 19/737 = CCM^*b*^ 9023	Jul 2019	Prague	Wound pus	M	32 y	Surgical wound infection	*S. aureus*
NRL/St^*a*^ 18/288 = P12563	Mar 2018	Lyon	Wound pus	M	51 y	Leg wound infection	NA^*e*^
NRL/St^*a*^ 21/187 = P13332	Jul 2021	Karlovy Vary	Bile	M	83 y	Cholelithiasis and cholecystitis	*E. coli, Klebsiella pneumoniae, Citrobacter freundii,* and *Enterococcus faecalis*
NRL/St^*a*^ 22/194 = P13326	Apr 2022	Prague	Swab of ear	M	6 m	Acute otitis media	Monoculture

^*a*^ NRL/St, National Reference Laboratory for Staphylococci, National Institute for Public Health, Prague.

^*b*^ CCM, Czech Collection of Microorganisms.

^*c*^ LMG, Bacteria Collection at the Laboratorium voor Microbiologie Universiteit Gent.

^*d*^ DSM, German Collection of Microorganisms and Cell Cultures.

^*e*^ NA, not analyzed.

The whole-genome phylogenetic distance from the related staphylococcal taxa with an ANI value of <92.3% (Table S3) and digital DNA-DNA hybridization (dDDH) of <45.6% determined the closest species *S. petrasii*; however, the values were below the species delineation thresholds, which are 95–96% and 70%, respectively (24). The whole-genome phylogeny thus confirmed that the five isolates represent a distinct *Staphylococcus* species designated *Staphylococcus brunensis* sp. nov.

### Growth, morphological, biochemical, and chemotaxonomical characterization of analyzed isolates

All five isolates exhibited Gram-positive stain, irregular cells ranging in diameter from 433 nm to 1,210 nm (Fig. S1) arranged in pairs, tetrads, and clusters. They grew very well on tryptone soy agar (TSA), Columbia agar with blood, plate count agar, P agar, and nutrient agar, and did not grow in a thioglycollate medium. The Congo red agar method showed negativity in the production of polysaccharide intercellular adhesin (PIA) associated with biofilm formation. Phenotypic identification based on bacitracin resistance and sensitivity to furazolidone, positive catalase test, growth in the presence of NaCl above 10%, and microscopic morphology assigned five isolates as *Staphylococcus* sp. In contrast to the main characteristics of staphylococci, isolate NRL/St 19/737 exhibited atypical negative catalase activity. Test-dependent results were also observed for the Voges-Proskauer (VP) test (acetoin) and for β-glucuronidase. With detection of acetoin production, we only obtained positive results in all five strains when pyruvic acid served as substrate using the commercial VP test (Erba Lachema) instead of glucose as substrate for the conventional VP test. Similar results were obtained with the β-glucuronidase test, where all isolates were positive in the STAPHYtest 24 kit, but negative in the API ZYM kit due to a different substrate for enzyme detection. The differentiation of novel staphylococcal isolates from similar and/or closely related staphylococci occurring in human clinical material is shown in Table 2. The species *S. petrasii*, *S. pragensis,* and *S. haemolyticus* were phenotypically the most similar taxa to the aforementioned isolates. Strain-dependent utilization results are specified in Table S4. Complete characteristics of *S. brunensis* sp. nov. are stated in the protologue given subsequently.

**TABLE 2:** Differentiation of *Staphylococcus brunensis* sp. nov. from closely related staphylococci occurring in human clinical material

Test^*d*^	Result obtained for indicated type strain^*a*^/result from species description						
	*S. brunensis* sp. nov.^*b*^	*S. petrasii* CCM 8418^T^	*S. croceilyticus* CCM 8421^T^	*S. pragensis* CCM 8529^T^	*S. haemolyticus* CCM 2737^T^	*S. borealis* CCM 9145^T^	*S. taiwanensis* CCM 9267^T^
Arginine dihydrolase	+	+/+	+/+	−/−	+/+	+/+	+/+
Voges-Proskauer test	+	+/+	+/+	+/+	w/+	−/−	+/+
Urease	−	+/d	+/+	−/−	−/−	+/+	+/+
β-Glucuronidase^*c*^	+	−/−	+/+	−/−	+/d	+/d	−/−
DNA hydrolysis	−	w/d	−/−	+/+	+/d	w/−	−/−
Acid from: lactose	+	−/d	−/−	−/−	−/d	−/−	−/−
Galactose	+	−/d	−/−	−/−	+/d	−/nt	−/−
Mannose	−	+/+	−/−	−/−	−/−	−/d	−/−
Ribose	−	−/w	w/w	−/−	−/d	+/+	+/+
D-arabinose	−	−/−	+/+	−/−	−/−	−/−	−/−
*N*-Acetylglucosamine	−	−/−	−/−	−/−	+/+	+/d	−/−
Pale yellow pigment	−	−/−	+/+	−/−	-/-	+/+	−/−

^*a*^ All data were taken from this study (in two replications).

^*b*^ Presented data are uniform for all isolates of *S. brunensis* sp. nov.

^*c*^ STAPHYtest 24 kit.

^*d*^ +, positive; -, negative; w, weak reaction; d, 11–89% strains positive; nt, not tested.

Antibiotic susceptibility testing showed that all five strains are susceptible to cefoxitin, clindamycin, gentamicin, chloramphenicol, linezolid, oxacillin, rifampicin, tobramycin, trimethoprim, sulphamethox/trimethoprim (cotrimoxazole), tetracycline, and fusidic acid. Susceptibility to ciprofloxacin and levofloxacin was intermediate. Susceptibility to ampicillin, penicillin G, tigecycline, and erythromycin was strain-dependent (Table S4).

**Fig 2:**
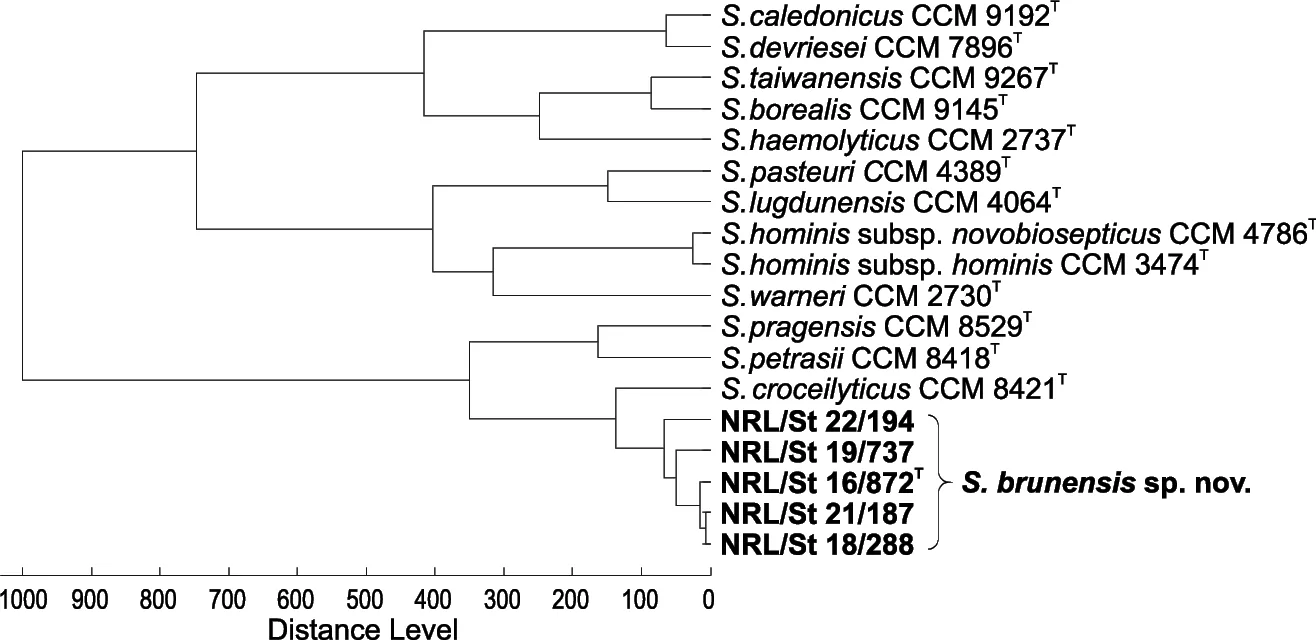
Dendrogram based on MALDI-TOF MS profiles of *Staphylococcus brunensis* sp. nov. and other phylogenetically related species. The dendrogram was generated using the correlation distance measure with the average linkage algorithm (UPGMA) settings of the software BioTyper version 3.1 (Bruker Daltonics).

By using cluster analysis of matrix-assisted laser-desorption/ionization-time-of-flight mass spectrometry (MALDI-TOF MS) protein profiles, all five *S*. *brunensis* sp. nov. strains were separated into a coherent cluster distinguished from phylogenetically related *Staphylococcus* spp., as shown in Fig. 2. All five strains share 35 signals within the *m/z* range 2–11 kDa, while five of these signals (*m/z* = 4130, 6660, 7614, 8258, and 10625) were found to be specific for *S. brunensis* sp. nov., being absent in the MALDI-TOF MS protein profiles of a comprehensive set of 65 *S*. *petrasii*, *S. croceilyticus*, and *S. pragensis* strains analyzed previously (10).

The chemotaxonomic analyses of type strain *S. brunensis* NRL/St 16/872^T^ showed predominantly menaquinone-7 (MK-7, 95%) and a small amount of MK-6 (4%) and MK-8 (1%). The major fatty acids were C _15:0 anteiso_ (38.6%) and C _17:0 anteiso_ (19.6%), followed by C _19:0 anteiso_ (8.3%), C _15:0 iso_ (6.3%), C _17:0 iso_ (6.9%), C _18:0_ (6.8%), C _19:0 iso_ (4.4%), and C _20:0_ (3.0%). A small amount of C _16:0 iso_ (1.8%), C _16:0_ (1.2%), and C _18:0 iso_ (1.6%) fatty acids were also present. The detected peptidoglycan type is A3α (L-Lys-Gly_3-4_, A11.2). All chemotaxonomic data are in-line with previous reported patterns (7).

### DNA fingerprinting of *S. brunensis* sp. nov.

**Fig 3:**
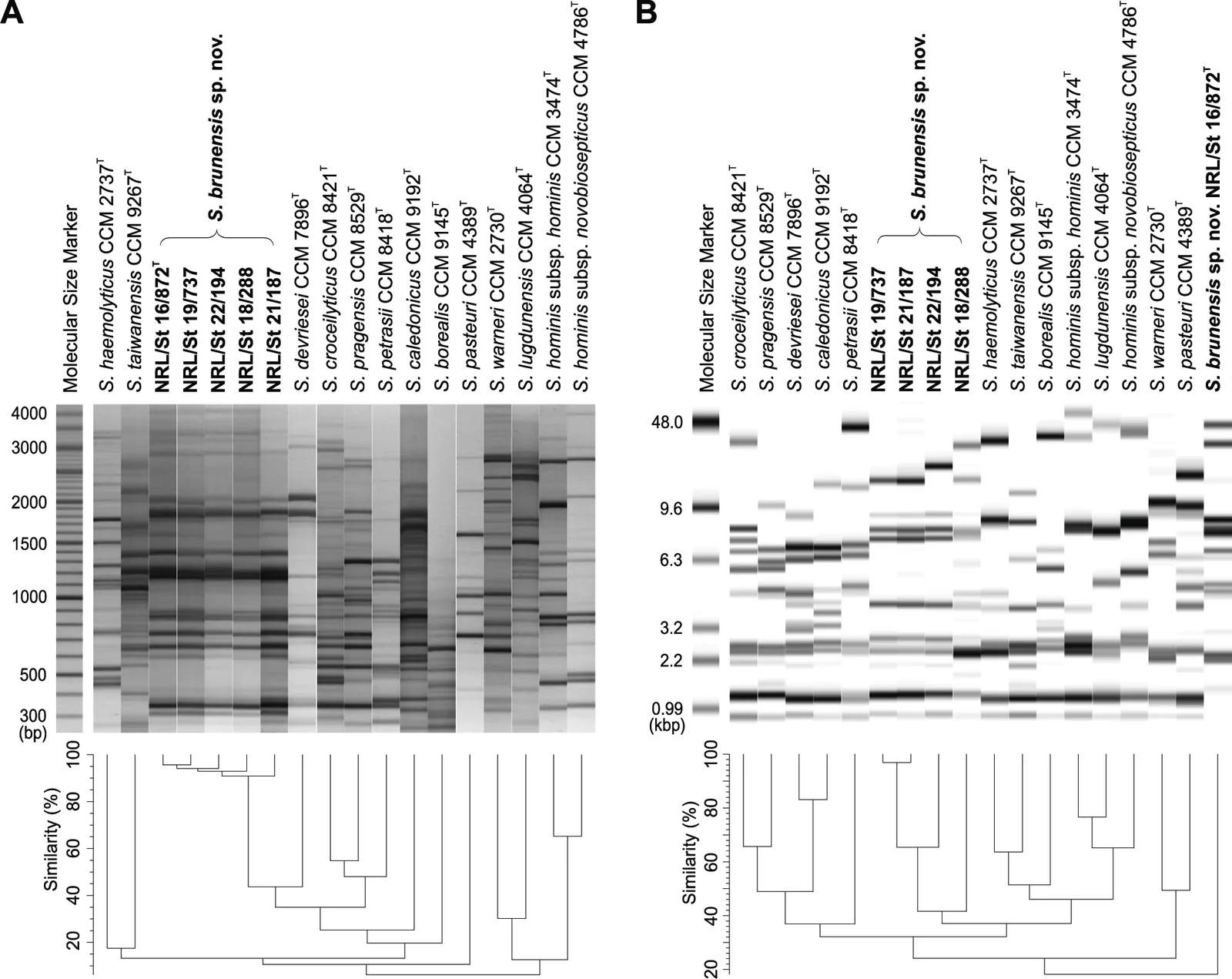
Cluster analysis of rep-PCR fingerprints and ribotype patterns obtained from *Staphylococcus brunensis* sp. nov. strains and the type strains of phylogenetically closely related *Staphylococcus* species. (**A**) Dendrogram based on (GTG)_5_-PCR fingerprints. (**B**) Dendrogram based on cluster analysis of *Eco*RI ribotype patterns obtained using a RiboPrinter system. The dendrograms were calculated with Pearson’s correlation coefficients with the UPGMA clustering method (*r*, expressed as percentage similarity values).

Screening of the investigated bacterial group by rep-PCR fingerprinting with primer (GTG)_5_ showed their genotypic coherence. All five isolates had visually similar fingerprints, which were grouped into a single cluster and separated from the other entries in the in-house fingerprint database, which includes members of all recognized species of the genus *Staphylococcus*, including type strains of phylogenetically closely related species (Fig. 3A). The obtained results also suggest that this rapid and easy-to-perform method can be used for the identification of *S. brunensis* sp. nov., similarly to how we demonstrated for the identification of *Staphylococcus* spp. in our previous studies (8, 23, 25).

In contrast to the rep-PCR method, automated ribotyping with the restriction enzyme *Eco*RI revealed heterogeneity among the five examined strains (Fig. 3B). Three isolates, NRL/St 16/872^T^, NRL/St 22/194, and NRL/St 18/288, exhibited unique fingerprint patterns that allowed differentiation at the strain level and indicated that they were not clonally related. The remaining two strains, NRL/St 19/737 and NRL/St 21/187, had visually identical ribotypes, despite being isolated in 2019 and 2021, respectively, at different locations and were therefore not considered clonally related. These results suggest that automated ribotyping with *Eco*RI can separate isolates of *S. brunensis* sp. nov. at the strain level, although some strains may have similar ribotype patterns. However, a reliable assessment of the discriminatory power of this technique for typing *S. brunensis* sp. nov. requires the analysis of a more significant number of strains from different localities and sources.

### Genome characterization of *S. brunensis* sp. nov.

**Fig 4:**
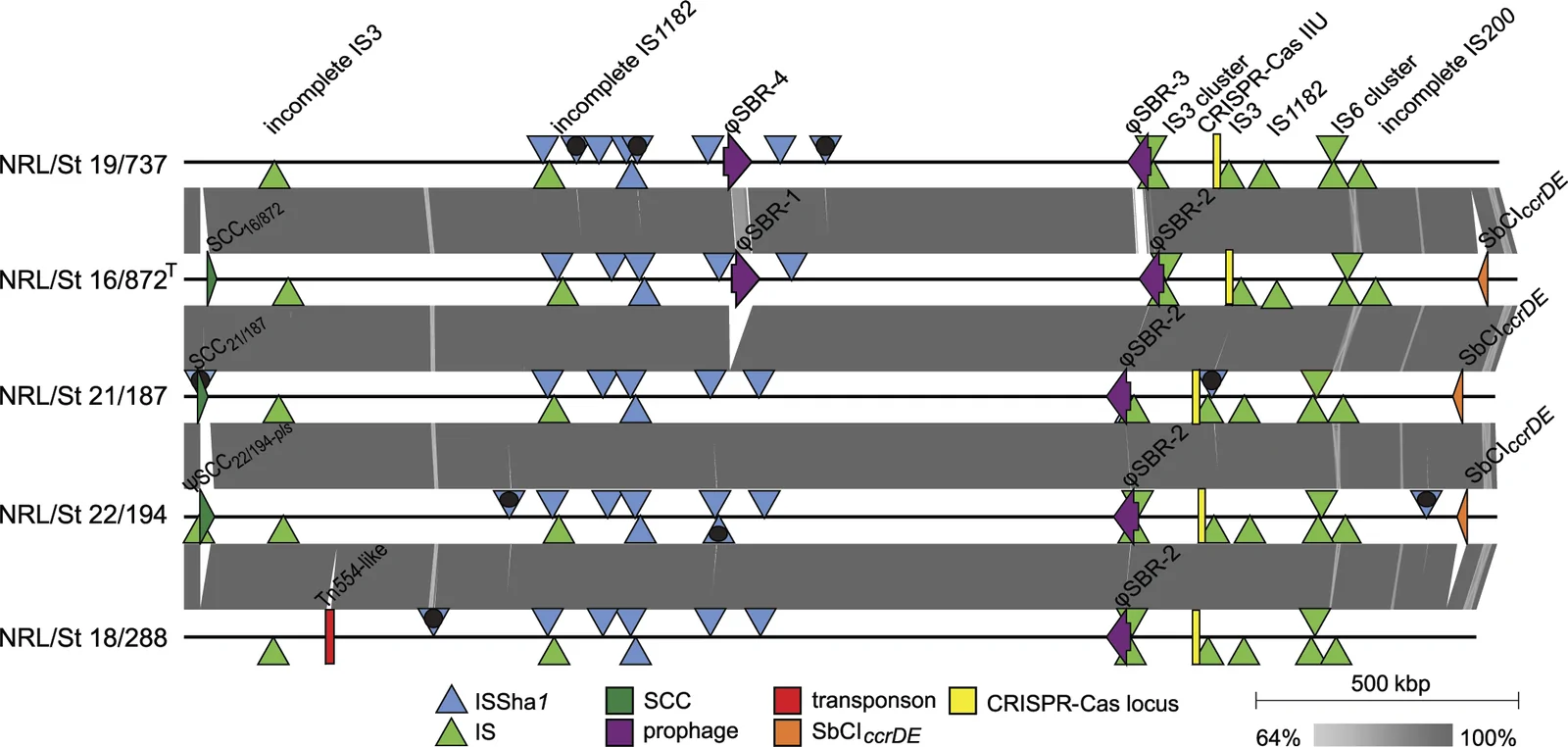
Genome comparison of *Staphylococcus brunensis* sp. nov. isolates. Mobile genetic elements are shown and color coded as in the legend. The ISSha*1* loci that are divergent for the respective strains are marked with black circles. Only nucleotide blast hits above 64% identity and longer than 2 kb are shown.

The comparison of sequenced genomes (Table S5) revealed a high degree of similarity between the isolates (Fig. 4). The *S. brunensis* sp. nov. genomes were 2.5–2.6 Mb long with GC content 33.3–33.4% encoding 2,500–2,700 CDS, 61‒62 tRNAs, and 19 rRNAs. The pangenome consists of 2,230 core and 416 accessory, and 569 unique genes in total. The sequenced genomes differ in variable genomic elements, which constitute 5–8% of the genome and are associated with virulence and antimicrobial resistance genes, predominantly located at plasmids. All *S. brunensis* sp. nov. genomes possess a type IIU CRISPR-Cas complex with 15–20 variable spacers, some of which target siphoviral prophages as determined by blastn search. The core gene *kat* encoding catalase in the strain NRL/St 19/737 (locus tag MT339_07575) has a 1 bp deletion in homopolymer polyA, which introduces a premature stop codon at the 3' end of the gene. It is possible that the catalase-negative phenotype of the strain is associated with the loss of function of this gene, although a truncated protein without the first 22 amino acids could be produced.

The *S. brunensis* sp. nov. genomes harbor several large plasmids, which can be grouped by the presence of either *rep39* or *rep20* gene. Due to high sequence variability and mosaicism caused by interspersed IS*431*, it was not possible to determine a complete consensus sequence for all plasmids. The plasmid contigs comprise beta-lactam, heavy metal, and disinfectant resistance as well as virulence and saccharide utilization genes as shown in Table 3. All analyzed strains harbor a small *rep21* plasmid conferring cadmium resistance. The strain NRL/St 22/194 contains an additional *rep10* plasmid harboring the *ermC* gene similar to the pUSA05-1 plasmid from *S. aureus* (26). These two small plasmids are integrated into the *rep39*-type plasmid contig of strain NRL/St 19/737. Furthermore, two cryptic plasmids, one containing the *rep13* gene and the other containing *rep5b* gene were identified (Table 3).

**TABLE 3:** Plasmid contigs of *Staphylococcus brunensis* sp. nov. grouped by replication gene and gene content

Replication genes	Strain	Size (kb)	Antimicrobial resistance	Virulence factors	Saccharide utilization genes	Mobilization/toxin-antitoxin systems
*rep39*	NRL/St 16/872^T^	31.2	*copZ*, *csoR*, *czrB*, *qacR*, *qacA*	*clp*	-	*mobA*
	NRL/St 18/288	21.9	*copZ*, *csoR*, *arsM*, *qacR*, *qacA*,	-	-	*fstP*
	NRL/St 21/187	22.9	*copZ*, *copA*, *csoR*, *arsR*, *arsM*	*clp*	-	*mobA*
*rep20*	NRL/St 16/872^T^	27.2	*arsR*, *arsM*	*isaB*, *essG*	-	*mobC*, relaxaseP, *mobP*
	NRL/St 19/737 and NRL/St 18/288	33.9	*arsR*, *arsM*	*isaB*, *essG*	*mqo*, *hxlR*, *hxlA*, *hxlI*	*mobC*, relaxaseP, *mobP*
	NRL/St 21/187	22.9	*qacR*, *qacA*	*isaB*, *essG*	-	
*rep39*, *rep5b*, *rep21*, *rep10*, *rep20*	NRL/St 19/737	32.0	*blaZ*, *blaR*, *blaI*, *ermC, cadX*, *cadA*, *cadD*	-	-	*mobC*, relaxaseP, *mobP*
*rep21*	NRL/St 16/872^T^, NRL/St 18/288, NRL/St 21/187, and NRL/St 22/194	2.8	*cadD*, *cadX*	-	-	-
*rep13*	NRL/St 16/872^T^ and NRL/St 21/187	2.6	-	-	-	-
*rep5b*	NRL/St 18/288	7.9	-	-	-	*mobC*, relaxase P
*rep10*	NRL/St 22/194	44.7	*ermC*			

The resistance to penicillin in *S. brunensis* sp. nov. NRL/St 18/288 is encoded by the *blaZ* gene located at a 9 kb Tn*554*-like transposon (Fig. 4) integrated into the chromosomal *isaB* gene. The chromosomes of *S. brunensis* sp. nov. harbor several copies of various full and partial IS elements from the IS*3*, IS*6*, IS*30*, IS*1182*, IS*200*/IS*605*, and ISha*1* families (Fig. 4). The IS*431* flanked composite transposon localized downstream of the *cspC* gene contains the gene for a short-chain dehydrogenase associated with survival in stress conditions (27). Each strain carries six to eight ISSha*1* copies integrated in six conserved and six variable loci, usually adjacent to rRNA genes. One copy of ISSha*1* is inserted in the SCC element of *S. brunensis* sp. nov. NRL/St 21/287 strain (Fig. 4).

The *S. brunensis* sp. nov. strains have one or two complete prophages in their genomes integrated into two different 18 bp *att* sites. The ΦSBR-1 prophage of NRL/St 16/872^T^ is integrated in the *att* site AATCCCTTACTTCCCGTT, located in the tRNA-Ser(gga) gene. The other strains encompass one or two CRISPR spacers homologous to the ΦSBR-1, and thus are immune to infection by this phage. The same *att* site is used by ΦSBR-4 in the genome of NRL/St 19/737. Both prophages are 45.4 kb long and share 79.3% nt identity. The next *att* site, AATCCCTCCGTTTCCGTT in tRNA-Ser(gct), is occupied by either ΦSBR-2 or ΦSBR-3. Prophage ΦSBR-2, which is 47 kb long, is integrated into the genomes of strains NRL/St 16/872^T^, NRL/St 19/288, and NRL/St 21/287. The strain NRL/St 19/737 harbors a 43.5 kb ΦSBR-3 prophage, which shares 79% nt identity with ΦSBR-2 genome along 57% of its length; the difference is mainly in the morphogenesis module. Both ΦSBR-2 and ΦSBR-3 carry a putative accessory virulence factor sialic acid transporter (28) in the lysis gene module.

Two *S*. *brunensis* sp. nov. strains NRL/St 16/872^T^ and NRL/St 21/287 comprise an approximately 18.2 kb-long genomic island in the rRNA methyltransferase H gene *rlmH* (*orfX*). The genomic island was almost identical in both strains; the only difference is the presence of an insertion sequence ISSha*1* in strain NRL/St 21/287. The island is bordered by imperfect 18 bp-long direct repeats GAAGC(A/G)TATCATAA(G/A)TGA and harbor *ccrA1B2* genes; thus, according to the rules of the IWG-SCC (29) the element was designated SCC_NRL/St 16/872_. The most similar element to SCC _NRL/St 16/872_ is a composite island in genome of *S. hominis* C34847 (30), sharing 95.2% nt identity along 75% of their length. The SCC_CCM9024_ carries genes for the type I restriction-modification (RM) system, sharing high homology with genes encoding a restriction (*hsdR*) and methylation (*hsdM*) subunit with the RM system of *S. hominis* C34847 and *S. aureus* NTUH-4729 (31). Interestingly, the *putP* gene, which encodes a sodium proline symporter, was wedged between the *ccrB2* gene and a set of three short hypothetical genes conserved in the *ccr* gene complex, thus disrupting the canonical organization of the complete *ccr* gene complex. Similarly, the *putP* gene and the flanking sequence from the *ccr* gene complex were found in SCC elements of various CoNS, *S. epidermidis* I1PPP121 (GenBank accession no. MH188479), *S. haemolyticus* BC05211 (KX181861), *S. hominis* J6 (LT963442), and *S. hominis* J11 (LT963438), and in plasmids of *S. warneri* 16A (CP031267) and *S. pasteuri* 3C (CP031281), pointing to a high degree of recombination occurring in staphylococci.

The strain NRL/St 22/194 also harbors an SCC element inserted into the *rlmH* gene bordered by the 21 bp imperfect repeat GG(C/A)GAAGC(A/G)TATCATAA(G/A)GTGA. The 26.6 kb pseudo-SCC element named ψSCC_NRL/St 22/194-*pls*_ has no recombinase genes, but carries several virulence genes, including the gene encoding plasmin sensitive protein (*pls*), poly(glycerol-phosphate) α-glycosyltransferase (*tagE*), and UDP-N-acetylmuramate-L-alanine ligase (*murF*), which are also found in *S. aureus* composite island SCC*mec*_WAMRSA40_ (32).

### Novel genomic island harboring cassette chromosome recombinase genes *ccrDE*

The strains NRL/St 16/872^T^, NRL/St 21/187 and NRL/St 22/194 harbor a mobile element of size 18.8 kb with cassette chromosome recombinase genes, integrated in the *rimJ/rimL* gene orthologous to the the ribosomal-protein-serine acetyltransferase gene *rimL* in *Escherichia coli* and *ydaF* in *Bacillus subtilis* (UniProt accession no. P13857 and P96579, respectively). The island designated *S. brunensis* chromosomal island *ccrDE* (SbCI*_ccrDE_*) harbors another homologue of *rimL* with 70% nt identity to the original *rimL*; thus, it may complement the function of the truncated *rimL* gene. SbCI*_ccrDE_* is bordered by the tetranucleotide motif GAAA. Additionally, the 19 bp direct repeat ATTCCACAATGAAATCCAT was found on the integration site in *rimL* and inside the *rimL* homologue on the island, which suggests a complex recombination event.

**Fig 5:**
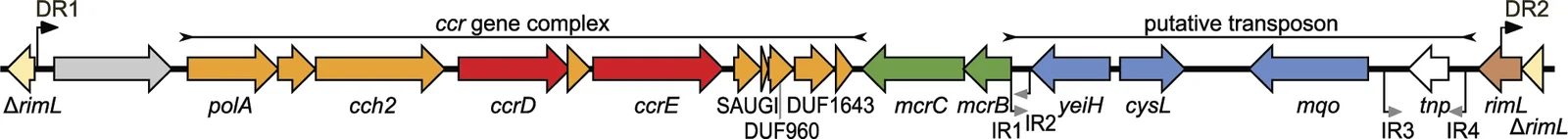
Annotated map of genes and possible functions in genomic island SbCI*_ccrDE_* harbored by *Staphylococcus brunensis* sp. nov. Genes are labeled according to known or putative function. Sequence of direct repeats DR1, DR2: 5′-ATTCCACAATGAAATCCAT-3′; sequence of inverted repeats IR1‒IR4: 5′-TGGTTCTGTTGCAAAGT-3′.

SbCI*_ccrDE_* possesses a cluster of genes homologous to the *ccr* gene complex from SCC elements. The core of the *ccr* complex consists of two *ccr* genes, similar to SCC elements type IV or II. Additional genes in the *ccr* complex, that is the putative primase *polA*, cassette chromosome helicase *cch2*, three short proteins with the domains SAUGI, DUF960, and DUF1643, and a hypothetical protein (Fig. 5), were homologous to those in SCC*mec* type V, which has only one *ccrC* gene in the *ccr* complex. SbCI*_ccrDE_ ccr* genes share more than 98% DNA sequence identity to *ccrA8B9* recombinases (Table S6) discovered recently in the *S. haemolyticus* genome (33). The values of nucleotide identity to currently known *ccrA1-7*, *ccrB1-8,* and *ccrC1-2* genes from SCCs range from 38.0 to 53.3% (Table S6). Although it is slightly above the threshold of 50.0% for the definition of a gene variant according to the rules of IWG-SCC (29), the amino acid (aa) identity level of SbCI*_ccrDE_* recombinases to known CcrA, CcrB, and CcrC reaches a maximum of 39.9%, which is substantially lower than aa identity among Ccr allotypes, which range from 50.9 to 92.3%. Therefore, based on the borderline nucleotide identities and low protein identities to CcrA, CcrB, and CcrC allotypes, we propose designating the SbCI*_cc_*_r_ recombinases as new allelic types *ccrD* and *ccrE* and reclassifying the described *ccrA8B9* recombinases accordingly (33). The CcrDE recombinases are able to excise the SbCI*_ccrDE_* element from its insertion sequence site, which has been detected by sequence read alignment and PCR analysis (Fig. S2).

Apart from the *ccr* gene complex, the island SbCI*_ccrDE_* carries the 5-methylcytosine-specific restriction enzyme *mcrB* and specificity subunit *mcrC* genes for the putative RM system (Fig. 5), commonly found on SCC elements, chromosomal islands, and prophages that help to maintain the mobile element in the genome (34). Approximately 5.8 kbp of the SbCI*_ccrDE_* is occupied by a putative transposon bordered by a perfect 17 bp inverted repeat of TGGTTCTGTTGCAAAGT. The transposon has one copy of the insertion sequence from the IS*6* family (sharing 95% nt identity to IS*431_mec_*) and accessory genes encoding putative membrane protein (*yeiH*), putative transcriptional regulator (*cysL*), and malate chinonine oxidoreductase (*mqo*) (Fig. 5), which are frequently found on plasmids.

**Fig 6:**
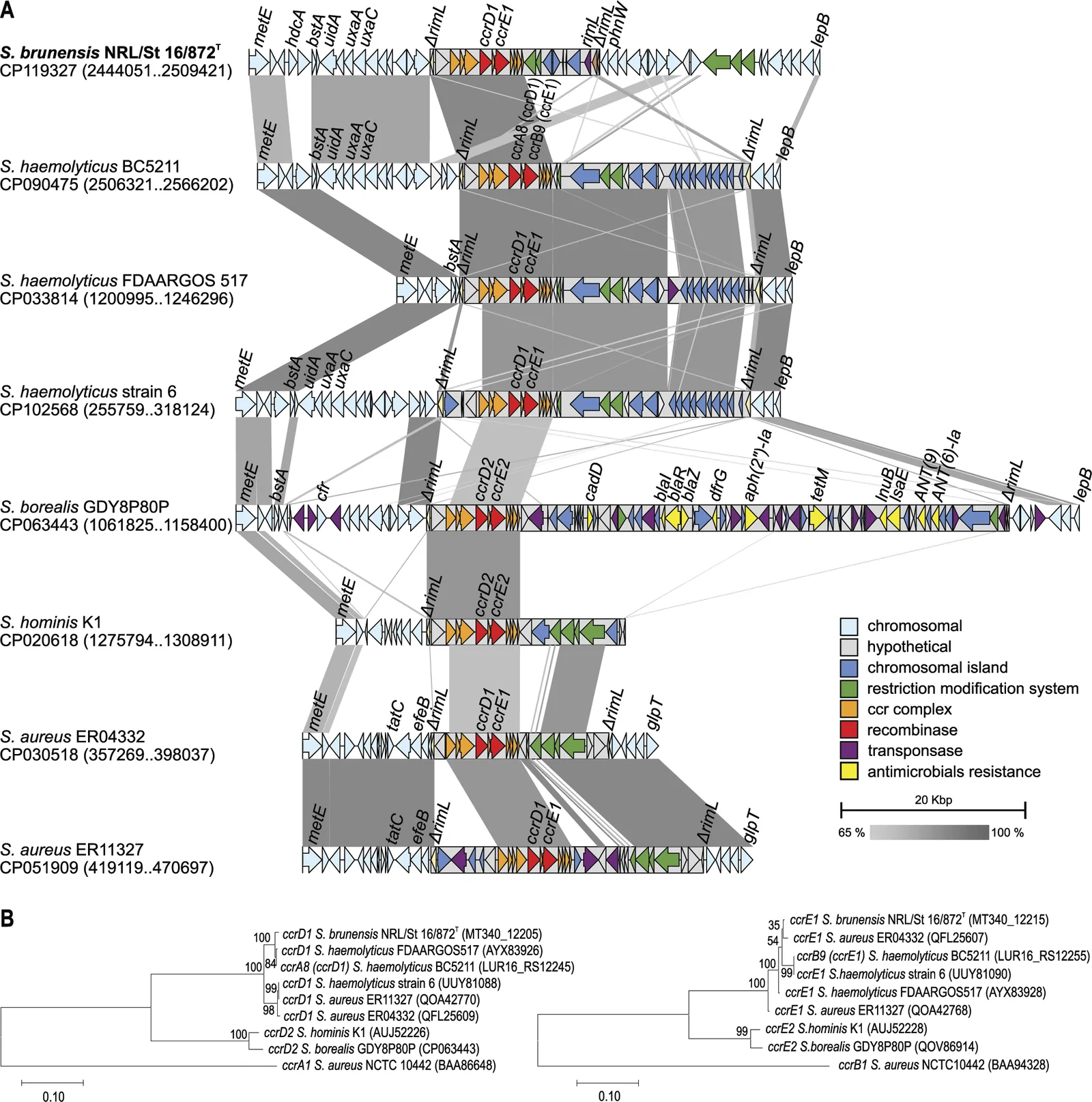
Comparative analysis of chromosomal islands harboring *ccrDE* cassette chromosome recombinases (CI*_ccrDE_*). (**A**) Comparison of CI*_ccrDE_* and flanking regions from different staphylococcal species. The genomic island CI*_ccrDE_* is highlighted with a gray background. Genes are labeled according to known or putative functions, as shown in the legend. Only nucleotide blast hits above 65% identity and longer than 2 kb are shown. The position of the island is provided in parentheses next to the GenBank accession number. (**B**) Maximum likelihood trees of nucleotide sequences of *ccrD and ccrA8* recombinases with *ccrA1* as an outgroup, and *ccrE and ccrB9* recombinases with *ccrB1* as an outgroup, respectively. The trees were constructed using Tamura-Nei model with 500 bootstrap replicates. The evolutionary distances are in the number of base substitutions per site. The protein GenBank accession numbers or locus tags are shown in parentheses.

We surveyed the GenBank database for sequences resembling SbCI*_ccrDE_*. In addition to *S. haemolyticus* BC5211 (33), we found related genomic islands with *ccrDE* in the genomes of *S. haemolyticus* (GenBank accession nos. CP033814 and CP102568), *S. hominis* subsp. *hominis* K1 (CP020618), and *S. borealis* GDY8P80P (35), and in *S. aureus* ER04332 and ER11327 (36), and related clones. The island is consistently inserted in the *rimL* gene, in a locus downstream of the conserved *metE* gene (Fig. 6A), which is 17–56 kb counterclockwise from the replication origin in CoNS species. However, in *S. aureus* strains, the *rimL* gene is located at the end of the *oriC* environ due to large-scale chromosomal inversion. The variable region of the islands comprises genes for an RM system (Fig. 6A). In *S. borealis* GDY8P80P*,* the island harbors many transposons with resistance genes to beta-lactam antibiotics, tetracycline, aminoglycosides, and other antimicrobials. The phylogenetic analysis of *ccrD* and *ccrE* genes from the related genomic islands (Fig. 6B) and the pairwise nucleotide identity comparison (Table S6) clearly distinguished two allotypes designated *ccrD1E1*, present in the genomes of *S. brunensis* sp. nov., *S. haemolyticus*, and *S. aureus*, and *ccrD2E2*, found in the genomes of *S. borealis* and *S. hominis* (Fig. 6B; Table S6).

### Taxonomic description of *S. brunensis* sp. nov.

*Staphylococcus brunensis* (*bru.nen′sis* L. adj. brunensis from Bruna, the Roman name of the city of Brno, Czech Republic, where this and other staphylococcal species were first described).

Cells are Gram stain positive cocci, occurring predominantly in pairs and clusters, non-spore-forming, and nonmotile. Colonies on TSA agar are circular, whole margin, flat, smooth, shiny, 2 mm in diameter, aerobic, and white. Hemolytic activity on sheep blood agar, production of delta-hemolysin revealed in synergistic test with a beta-hemolytic producing strain (*S. pseudintermedius* CCM 4710). Growth in the presence of 12% NaCl, at 20°C and 45°C, but not at 15°C and 48°C. They are positive for pyrrolidonyl arylamidase, arginine dihydrolase, and nitrate reduction and negative for coagulase, clumping factor, oxidase, urease, VP test (acetoin, conventional tube test), hyaluronidase, thermostable nuclease, and ornithine decarboxylase; susceptible to furazolidon (100 µg) and novobiocin (5 µg), and resistant to bacitracin (10 IU); partially resistant to lysostaphin (200 mg L^−1^) and resistant to lysozyme; negative for hydrolysis of esculin, gelatine, DNA, and Tween 80; positive on API ZYM for acid phosphatase, alkaline phosphatase (weak), esterase (C 4), and esterase lipase (C 8); negative on API ZYM for lipase (C14), valine arylamidase, cystine arylamidase, trypsin, α-chymotrypsin, naphthol-AS-BI-phosphohydrolase, α-galactosidase, β-galactosidase, β-glucuronidase, β-glucosidase, *N*-acetyl-β-glucosaminidase, α-mannosidase, and α-fucosidase. *S. brunensis* sp. nov. produce acid from glycerol (weak), galactose, D-glucose, D-fructose, maltose, lactose, sucrose, and trehalose. They do not produce acid from erythritol, D-arabinose, L-arabinose, ribose, D-xylose, L-xylose, adonitol, β-methyl-D-xyloside, mannose, sorbose, rhamnose, dulcitol, inositol, mannitol, sorbitol, α-methyl-D-mannoside, α-methyl-D-glucoside, *N*-acetyl glucosamine, amygdaline, arbutine, salicin, cellobiose, melibiose, inulin, melezitose, D-raffinose, starch, glycogen, xylitol, β-gentiobiose, D-turanose, D-lyxose, D-tagatose, D-fucose, L-fucose, D-arabitol, L-arabitol, gluconate, 2-keto-gluconate, and 5-keto-gluconate.

Variable biochemical reactions were obtained for catalase (4 of 5 positive), leucine arylamidase (2 of 5 positive), α-glucosidase (1 of 5 positive), and growth in 15% NaCl (4 of 5 positive) (Table S4).

Utilization (Biolog MicroPlate GEN III, protocol A) is positive for D-maltose, D-trehalose, D-turanose, α-D-glucose, D-fructose, D-galactose, glycerol, L-alanine, L-arginine, L-glutamic acid, L-serine, D-gluconic acid, acetic acid, and formic acid. Negative utilization for D-cellobiose, gentiobiose, stachyose, D-raffinose, D-melibiose, β-methyl-D-glucoside, D-salicin, *N*-acetyl-D-glucosamine, *N*-acetyl-D-galactosamine, *N*-acetyl neuraminic acid, 3-methyl glucose, D-fucose, L-fucose, L-rhamnose, inosine, D-sorbitol, D-mannitol, myo-inositol, D-glucose-6-PO4, D-aspartic acid, D-serine, gelatin, glycyl-L-proline, L-histidine, D-galacturonic acid, D-galactonic acid lactone, D-glucuronic acid, glucuronamide, mucic acid, quinic acid, D-saccharic acid, *p*-hydroxy phenylacetic acid, D-lactic acid methyl ester, citric acid, α-keto glutaric acid, D-malic acid, bromo-succinic acid, γ-amino-butyric acid, α-hydroxy-butyric acid, β-hydroxy-D,L-butyric acid, and propionic acid.

The type strain is NRL/St 16/872^T^ (= CCM 9024^T^ = DSM 111349^T^ = LMG 31872^T^). The major respiratory quinone is menaquinone-7. The major fatty acids are C_15:0 anteiso_ and C_17:0 anteiso_. The peptidoglycan type is A3α (A11.2). The DNA G+C content of strain NRL/St 16/872^T^ is 33.40 mol%, calculated from the whole-genomic sequence. The species description is based on the characterization of five strains isolated from various human clinical materials. Most of the characteristics of the type strain NRL/St 16/872^T^ agree with the species description. The GenBank/ENA/DDBJ accession number for the 16S rRNA gene is OQ401401. The complete chromosome sequence of the type strain is available under GenBank accession number CP119327.

## DISCUSSION

The recent molecular diagnostic methods and polyphasic taxonomic approach, including whole-genome sequencing, allow for more effective species differentiation of CoNS from various sources. Here, we described *S. brunensis* sp. nov., which occupies similar niches to the closely related species from the *S. petrasii* phylogenetic complex (10). The available isolates of *S. brunensis* sp. nov. were associated with the human ear and wound infections, whereas *S. petrasii* and *S. pragensis* predominated in wounds, blood, or urinary tract infections (Table S7). However, it is challenging for clinicians to determine whether these CoNS are causative agents of human diseases or suspected contaminants associated with the occurrence of these commensal bacteria on human skin.

The pathogenic potential of CoNS is related to immune evasion, invasion of host tissues, and biofilm formation, allowing them to persist on the surfaces of indwelling medical devices and thus cause chronic infections (1). The intercellular adhesion operon (*ica*), biofilm-associated protein (*bap*/*bhp*), and fibronectin-binding protein genes (*fnbA*/*fbe*) directly associated with biofilm production in *S. epidermidis* (37) have not been identified in either *S. brunensis* sp. nov. genomes or the other species from *S. petrasii* complex. Likewise, the *ica* operon homologue has not been detected in *S. haemolyticus* (3) and may be missing in this phylogenetic clade. Biofilm formation in the initial phase is influenced by molecules involved in surface adhesion. In staphylococci, these are microbial surface components recognizing adhesion matrix molecules (MSCRAMMs) (38). Homologs of cell wall-anchored serine-aspartate repeat-containing protein genes *sdrC, sdrG*, and *sdrH* have been identified in *S. petrasii* genomes (10) but not in *S. brunensis* sp. nov. However, gene homologues for elastin-binding protein (*ebp*), thermonuclease (*nuc*), autolysin E (*atlE*), and gene clusters (*cap5* or *cap8*) involved in the synthesis of capsular polysaccharides have been found in genomes of both *S. petrasii* (10) and *S. brunensis* sp. nov.

A comparison of representative genome sequences revealed that the species *S. brunensis* sp. nov., *S. croceilyticus*, *S. petrasii*, and *S. pragensis* differ only marginally in genomic G+C content, genome size, and the median protein count encoded by the core genome. The major cause of interspecies differences in the *S. petrasii* complex is the accessory genome and variable genetic elements (Fig. S3). The role of these elements is best described in *S. aureus*, where HGT contributes to adaptation and evolution into successful lineages (39 – 41).

More than 90% of clinical staphylococcal isolates harbor plasmids of various size that can be classified to small multicopy plasmids or larger plasmids carrying several resistance determinants. However, only 5% (42) of staphylococcal plasmids are large multiresistance elements with the ability to mobilize or undergo conjugative transfer. Strains of *S. brunensis* sp. nov. harbor several large plasmids that encode genes for mobilization (*mobA*, *mobC*, *mobP*) and their spread by HGT is possible. The resistance genes are usually cointegrated between two copies of ISs that promote their spread (43). An example is IS*431* previously described in plasmids pSK41 and pGO1 (44), harboring linezolid and high-level resistance to vancomycin (45). It is probable that IS*431*, identified in *S. brunensis* plasmid sequences, is responsible for the mosaic structure of the elements and also promotes the spread of resistance genes across the genus *Staphylococcus*.

The plasmid-borne resistance to antibiotics and disinfectants suggests enhanced survival in healthcare environments. The resistance to beta-lactam (*blaZ*) and macrolide (*ermC*) antibiotics in *S. brunensis* sp. nov. strains correlates with resistance genes on plasmids and the Tn*554*-like transposon. Resistance to quaternary ammonium compounds (*qacA*), copper and heavy metals, and the hexulose utilization operon (*hxl*) in plasmids of *S. brunensis* sp. nov. suggests adaptation to various ecological niches similar to nosocomial and community-associated isolates of *S. haemolyticus* (46, 47).

The genomes of *S. brunensis* sp. nov. contain more insertion sequence elements than the closest relatives from *S. petrasii* complex (7, 8). The copy number variability of IS implies their recent capture and propagation and increases the genome plasticity among the strains. The most abundant ISSha*1* elements with 98% similarity to ISSha*1* from *S. haemolyticus* (3) are often located near rRNA genes and may contribute to variable ribotype patterns.

A significant MGE in the genome of *S. brunensis* sp. nov. is the genomic island SbCI*_ccrDE_* with unique recombinase genes *ccrD1E1*. Xiao et al. (33) reported the existence of these recombinases as *ccrA8B9* allotypes. Nevertheless, their amino acid sequences that were highly different from other known *ccrAB* variants place them among novel *ccrDE* alleles. This reclassification also made it possible to distinguish another apparent variant *ccrD2E2,* present in *S. hominis* and *S. borealis*. The *ccrDE* genes in all inspected staphylococcal genomes were present on a genomic island related to SbCI*_ccrDE_*. Chromosomal islands with *ccrDE* (CI*_ccrDE_* s) share gene structure similar to SCCs, such as conserved *ccr* complex, presence of variable regions, and flanking by direct repeats. However, unlike SCCs, which are canonicaly inserted in the *rlmH* (*orfX*) gene in the *oriC* environ (48), CI*_ccrDE_* s are inserted in the *rimL* gene. The SCC-like islands with Ccr or closely related large serine recombinase were inserted in the *rlmH* in other genera of Gram-positive bacteria, that is the genera *Mammaliicoccus* and *Macrococcus*, *Enterococcus faecium*, *Bacillus cereus*, *Geobacillus vulcani*, and *Clostridioides difficile* (49 – 52). Hence, CI*_ccrDE_* s might represent a new class of SCC elements integrated into the *rimL* gene due to the altered specificity of the *ccrDE* recombinases.

The molecular mechanism of the action of *ccrAB* recombinases requires specific sequences of around 60–70 bp and the presence of the central dinucleotide GA in the integration site, which are essential for the integration of the cassettes into the highly conserved *rlmH* gene (20). However, the *ccrAB* recombinases also act on non-canonical less conserved recombination sites (20). Xiao et al. (33) showed that the *ccrDE* recombinases are functional in excision and transfer of an SCC from *rlmH*, but they do not move together with the cassette. Here, we proved that the CI*_ccrDE_* bearing the *ccrDE* genes is excised from the chromosome. Since CI*_ccrDE_* has a similar size as pathogenicity islands and possesses a set of genes required for its replication (53), the element would perfectly fit into the phage capsid to be spread via transduction, which is a common transmission route of MGE in staphylococci (54, 55). The high sequence conservation of the *ccrDE* gene complex in various species corresponds with their spread through horizontal gene transfer.

The proportion and diversity of CI*_ccrDE_* was low in the sequenced genomes deposited in the public databases, making it extremely difficult to identify the donor of *ccrDE* complex responsible for the formation of this element. Since we found *ccrD1E1* and *ccrD2E2* predominantly in CoNS genomes, it is possible that CoNS species are predisposed to forming this element and it can subsequently be transferred to *S. aureus*. The variable regions of CI*_ccrDE_* are highly diverse in gene content, suggesting that the CI*_ccrDE_* element evolved only recently in independent acquisition events, which are also common in the evolution of SCC*mec* (56) or staphylococcal pathogenicity islands (57). The benefit of CI*_ccrDE_* for the bacterial host might be only marginal, so there is no selective pressure to maintain established element and disseminate it to more strains. The only widespread genes identified in all CI*_ccrDE_* s that help to preserve the element are the RM system genes that differ in the CI*_ccrDE_* s, suggesting that they come from various sources similar to canonical SCC elements (30, 31).

With the high selective pressure exerted on staphylococci by the use of antimicrobials, this element is a perfect candidate for the acquisition and spread of resistance and virulence genes. The SCC*mec* element underlying the successful spread of MRSA clones originated through joining of *ccr* gene complex with *mec* gene complex coming from multiple sources. The first signs of the accumulation of resistance genes in CI*_ccrDE_* were observed in *S. borealis*, previously misidentified as *S. haemolyticus* (35), where the island comprised several transposons. A similar cluster of drug-resistance genes to that in CI*_ccrDE_* of *S. borealis* was also reported in *S. aureus* on an SCC element (58) and a plasmid (59). We conclude that the gene structure of CI*_ccrDE_* indicates its ability to act as a primordial element to accumulate virulence and antimicrobial resistance factors. The spread of the island to established pathogens such as *S. aureus* would thus represent a new threat to the healthcare system.

### Conclusion

The identification of the new species *S. brunensis* within the *Staphylococcus* genus expands our understanding of the diversity of coagulase-negative staphylococci. The number of the strains available is still limited, but similar to its closest relative *S. petrasii*, it can be expected that more strains will be captured as better diagnostic methods are developed. Genome analysis of the new isolates has important implications for studying the role of coagulase-negative staphylococci as a reservoir of transmissible genes that can facilitate improved survival in the environment, resistance to antibiotic treatment, or increased virulence following horizontal transfer. Characterization of a previously unexplored genomic island closely related to the SCC indicates the potential for its interspecies transfer enabled by unique *ccrDE* recombinase genes in both coagulase-negative staphylococci and the more clinically significant *S. aureus*. Identifying the new type of MGE thus opens up new possibilities for future research of gene transfer mechanisms in the emergence of multidrug-resistant staphylococcal strains with implications for clinical practice. These findings deepen our understanding of the evolution and pathogenesis of staphylococci, shedding light on how these bacteria acquire and disseminate virulence traits and antibiotic resistance.

## MATERIALS AND METHODS

### Bacterial strains, cultivation and phenotypic identification, and antimicrobial susceptibility testing

Simultaneously analyzed reference strains of *Staphylococcus* spp. were obtained from the Czech Collection of Microorganisms (CCM, Masaryk University, Brno). The strains grew well in a basic set of staphylococcal media at a temperature of 30–37°C. The morphological, biochemical, and physiological characterization was performed as previously mentioned (7, 60 – 62). Antimicrobial susceptibility testing by disc diffusion method on Mueller-Hinton agar with adherence to EUCAST guidelines (63) was performed as described previously (60).

### Genome sequencing and bioinformatics analyses

The short-read sequencing was conducted for the type strain NRL/St 16/872^T^ (LGC Genomics, Berlin, Germany). The genomic DNA was isolated with a GenElute Bacterial Genomic DNA kit (Sigma-Aldrich, St. Louis, MO, USA) from pure culture colonies cultivated on Colombia sheep blood agar (Oxoid). The library was prepared using a Nextera XT DNA Library Preparation Kit (Illumina, San Diego, CA, USA) and sequenced externally by LGC Genomics (Berlin, Germany) on the NextSeq platform with 2 × 150 bp reads (Illumina). Genomes of all novel isolates were sequenced by Oxford Nanopore Technology (ONT). The genomic DNA was extracted as described previously (64). Sequencing libraries were prepared using an SQK-RAD004 rapid barcoding kit and sequenced with a FLO-FLG001 cell in a MinION device and MinKnow v21.10.4 software (Oxford Nanopore Technologies, Oxford, UK).

The software Guppy version 6.0.0 (Oxford Nanopore Technologies) with config dna_r9.4.1_450bps_sup.cfg and default settings was used for basecalling, demultiplexing, and barcode trimming. The ONT reads were filtered by quality mapping to Illumina reads using Filtlong version 0.2.1 (https://github.com/rrwick/Filtlong) with a minimum length of 1,500 bp and quality threshold set to 95% and mapping on Illumina reads where applicable. The quality of both long and short reads was assessed with FastQC version 0.11.9 (http://www.bioinformatics.babraham.ac.uk/projects/fastqc) and NanoStat (65). Complete chromosome and partial plasmid sequences were obtained using either a hybrid assembly with Unicycler version 0.4.9 (66) or a long-read-only assembly with Trycycler version 0.5.3 (67). The resulting contigs were further polished with Medaka version 1.6.1 (https://github.com/nanoporetech/medaka) and Polypolish version 0.5.0 (68). Sequences were manipulated and inspected in the cross-platform bioinformatics software Ugene version 38.1 (69). The genomes were annotated using the NCBI Prokaryotic Genome Annotation Pipeline (70). Nucleotide and protein multiple sequence aligment was performed with Clustal Omega (71). The multiple sequence alignment was visualized using EasyFig version 2.2.5 (72) and IslandCompare version 1.0 (73). Variable genetic content was identified with PhiSpy version 3.4 (74), MobilomeFinder (75), PlasmidFinder (76), IslandViewer 4 (77), ISFinder (78), and SCCmecFinder (79). The CRISPR/Cas system was characterized by CRISPRCasTyper (80). Virulence and resistance genes were predicted using Abricate (https://github.com/tseemann/abricate) with the CARD (81), Resfinder (82), and VFDB (83) databases.

### Phylogenetic and pangenomic analyses

The partial 16S rRNA gene was sequenced by Sanger sequencing in the Eurofins MWG Operon sequencing facility (Ebersberg, Germany) with previously described primers (84). Whole-genome sequences of related staphylococcal species were obtained from the NCBI database (Table S1). The multilocus sequence data of six housekeeping genes (*rpoB*, *groEL*, *dnaJ*, *tufA*, *sodA*, and *gap*) that are commonly used in phylogenetic studies of the *Staphylococcaceae* were extracted from whole-genome sequence assemblies as follows (gene coordinates of *S. aureus*): 1420..1974 for *rpoB*, 270..826 for *hsp60*, 23..911 for *dnaJ*, 49..929 for *gap*, 383..1032 for *tufA*, and 50..480 for the *sodA* gene. The phylogenetic analyses were performed with the software MEGA version 11 (85). The UBCG collection of 92 conservative genes (86), the average nucleotide identity (ANI) (87), and digital DNA-DNA hybridization (dDDH) values by the d4 formula using the web-based genome-to-genome distance calculator (GGDC) version 3.0 (88) were used for calculations of overall genome relatedness indices. The pangenome was calculated with the OrthoVenn2 pipeline with proteins clustered at the default threshold (89).

### DNA fingerprinting

For genotypic characterization of the investigated bacterial group, fingerprinting by repetitive sequence-based PCR (rep-PCR) with the primer (GTG)_5_ (90) and automated ribotyping with the restriction enzyme *Eco*RI were performed. The isolation of DNA for rep-PCR fingerprinting, PCR conditions, and fingerprint analysis were performed as previously described (91). Automated ribotyping was performed using the RiboPrinter microbial characterization system (DuPont Qualicon) according to the manufacturer’s instructions. Numerical analysis of rep-PCR fingerprints and *Eco*RI ribotype patterns was performed using BioNumerics version 7.6 (Applied Maths, Belgium). The ribotype patterns were imported into the BioNumerics software using the load samples import script provided by the manufacturer.

### PCR analysis of mobilizable genomic island encoding Ccr recombinases

To determine whether a genomic island is mobilizable, we designed primers spanning the excision site and primers targeting the key genes present in the genomic island (Table S2). The PCR reaction was conducted using Quick-Load 2× master mix with standard buffer (New England Biolabs, Ipswich, MA, USA) and a 200 nM concentration of each primer. The genomic island product was further analyzed by Sanger sequencing (Eurofins Genomics, Germany).

### Transmission electron microscopy

A 200-mesh carbon/formvar-coated grid was placed on a drop of suspension of bacteria in water for 20 min. Bacterial cells on the grid were negatively stained with 2% ammonium molybdate and treated with UV light. A Morgagni 268D Philips (ThermoFisher Scientific, The Netherlands) transmission electron microscope was used to visualize bacterial cells.

### MALDI-TOF MS

Protein fingerprinting by means of MALDI-TOF MS using an Ultraflextreme instrument (Bruker Daltonics, Germany) was conducted after a standard extraction protocol (92). MALDI-TOF mass spectra were obtained using an UltrafleXtreme instrument (Bruker Daltonics) operated in linear positive mode using the software FlexControl version 3.4. Signals present in at least seven out of nine independent mass spectra acquired per sample were taken into account. Mass spectra were processed using FlexAnalysis version 3.4 (Bruker Daltonics) and BioTyper version 3.1 software (Bruker Daltonics) supplemented with database version 10.0.0.0 (9,607 entries).

### Chemotaxonomic characterization

Respiratory quinones were extracted and analyzed as previously described (93). Identity was confirmed by mass spectrometry, as described by Schumann et al. (94). Analysis of the cellular fatty acid profile was performed using a Microbial Identification System (MIDI, Newark, DE) according to the Standard Protocol of the Sherlock Microbial Identification System software, version 6.1 (95). The fatty acids were identified using gas chromatography–mass spectrometry (GC-MS) according to the study of Vieira et al. (93).

Isolation and structural analysis of the peptidoglycan was performed according to published protocols with some modifications. Briefly, the amino acid composition of the total hydrolysate (4 N HCl, 100°C for 16 h) of the peptidoglycan was analyzed by GC/MS (protocol 10 by Schumann [96]). The partial hydrolysate (4 N HCl, 100°C, 0.75 h) of the peptidoglycan was analyzed by high-resolution liquid chromatography–mass spectrometry (LC-MS) as described previously (94, 96). Enantiomeric analysis was performed by liquid chromatography as described by reference 97.

## Data Availability

The complete genome sequences of new isolates NRL/St 16/872^T^, NRL/St 19/737, NRL/St 18/288, NRL/St 21/187, and NRL/St 22/194 have been deposited in GenBank/ENA/DDBJ database under accession numbers CP119327‒CP119331, JALGRI000000000, JALGRH000000000, JALGRG000000000, and CP116597‒CP116599, respectively. The associated BioProject number is PRJNA779217. The accession number for the 16S rRNA gene of the type strain NRL/St 16/872^T^ is OQ401401.

## SUPPLEMENTAL MATERIAL

Supplemental tables and figures spectrum.01342-23-s0001.pdf Tables S1 to S5, Table S7, Figures S1 to S3.

Table S6 spectrum.01342-23-s0002.xlsx Pairwise identity matrices of nucleotide and amino acid sequences of cassette recombinases.

ASM does not own the copyrights to Supplemental Material that may be linked to, or accessed through, an article. The authors have granted ASM a non-exclusive, world-wide license to publish the Supplemental Material files. Please contact the corresponding author directly for reuse.
