# Supplementary material for: Staphylococcus brunensis sp. nov. isolated from human clinical specimens with a staphylococcal cassette chromosome-related genomic island outside of the rlmH gene bearing the ccrDE recombinase gene complex
Source: Microbiol Spectr. 2023 Sep 15;11(5):e01342-23. doi: 10.1128/spectrum.01342-23 (PMC10581047; doi:10.1128/spectrum.01342-23)
Supplement: Supplemental tables and figures — Tables S1 to S5, Table S7, Figures S1 to S3. [file spectrum.01342-23-s0001.pdf]

Staphylococcus brunensis sp. nov. isolated from human clinical specimens with a staphylococcal cassette chromosome-related genomic island outside of the *rlmH* gene bearing the *ccrDE* recombinase gene complex

Vojtěch Kovařovic, Adéla Finstrlová, Ivo Sedláček, Petr Petráš, Pavel Švec, Ivana Mašlaňová, Meina Neumann-Schaal, Ondrej Šedo, Tibor Botka, Eva Staňková, Jiří Doškař and Roman Pantůček*

*** Correspondence:** Roman Pantůček: pantucek@sci.muni.cz

Supplementary Figures and Tables

Table S1. Whole-genome sequences of reference staphylococcal species used in phylogenetic analyses, obtained from NCBI Assembly database.

Strain	Assembly accession number
S. borealis 51-48^T	GCA_013345165.1
S. brunensis NRL/St 16/872^T	GCA_022815905.1
S. caledonicus H8/1^T	GCA_016238465.1
S. croceilyticus CCM 8421^T	GCA_004684875.1
S. devriesei NCTC13828^T	GCA_900458355.1
S. haemolyticus ATCC 29970^T	GCA_006094395.1
S. hominis subsp. *hominis* NCTC 11320^T	GCA_002901845.1
S. hominis subsp. *novobiosepticus* CCUG 42399^T	GCA_002902465.1
S. lugdunensis NCTC 12217^T	GCA_900478255.1
S. pasteuri DSM 10656^T	GCA_003970495.1
S. petrasii NCTC 13835^T	GCA_900458665.1
S. pragensis CCM 8529^T	GCA_014635505.1
S. taiwanensis NTUH-S172^T	GCA_020544305.1
S. warneri NCTC 11044^T	GCA_900636385.1

Table S2. List of primers for analysis of *Staphylococcus brunensis* sp. nov. chromosomal island *ccrDE* (SbCI$_{ccrDE}$) harbouring Ccr recombinases.

Construct detected	Forward primer		Reverse primer		Product size(bp)
Mobilized genomic island SbCI$_{ccrDE}$	SBCI_18056_F	CACACTCTGGACAAAATTTCACA	SBCI_751_R	GCCTGTGTCTCAAGTTGATTCA	1244
Sanger sequencing of mobilized genomic island	SBCI_18074_F	TCACAATATAACACTCCCCTT	SBCI_367_R	TATTTCAATTTGGAGTCGGAT	823
CcrD recombinase gene	ccrD_621_F	ACCCCTAAGCAAGTGATGCG	ccrD_846_R	GAATTGGCGTCATTGTTGCG	226
CcrE recombinase gene	ccrE_987_F	TGCCGAACACGTAAACAAAG	ccrE_1601_R	AGCCAATAAAGAAGACGAAC	615
attB of genomic island SbCI$_{ccrDE}$	rimL_246_F	AACCGATGACTCCTGCTAACG	rimL_39_R	ATTTTGGAAGAGCGTGAAGCG	208

Table S3. Heatmap table of average nucleotide identity (ANI) values generated from pairwise comparison of whole-genome sequences from *Staphylococcus brunensis* sp. nov. strains and phylogenetically related taxa of the genus *Staphylococcus*. An ANI score greater than 95–96% between two genomes indicates that they are members of the same species. The whole-genome sequences used are listed in Table S1.

Strain	*S. brunensis* NRL/St 16/872^T	*S. brunensis* NRL/St 19/737	*S. brunensis* NRL/St 18/288	*S. brunensis* NRL/St 21/187	*S. brunensis* NRL/St 22/194	*S. petrasii* NCTC 13835^T	*S. croceilyticus* CCM 8421^T	*S. pragensis* CCM 8529^T	*S. haemolyticus* ATCC 29970^T	*S. borealis* strain 51-48^T	*S. taiwanensis* NTUH-S172^T	*S. caledonicus* H8-1^T	*S. devriesi* NCTC 13828^T	*S. hominis* subsp. *hominis* NCTC 11320^T	*S. hominis* subsp. *novobiosepticus* CCUG 42399^T	*S. lugdunensis* NCTC 12217^T	*S. pasteuri* DSM 10656^T	*S. warneri* NCTC 11044^T
S. brunensis NRL/St 16/872^T	100	99.8	99.9	99.9	99.9	92.3	91.1	89.9	82.3	82.3	82.9	82.4	82.2	81.2	81.2	79.4	79.8	79.9
S. brunensis NRL/St 19/737	99.8	100	99.8	99.8	99.8	92.2	91.1	90.0	82.3	82.2	82.6	82.4	82.2	81.0	81.0	79.2	79.8	80.0
S. brunensis NRL/St 18/288	99.9	99.8	100	99.8	99.9	92.3	91.1	90.0	82.4	82.3	82.7	82.3	82.2	81.0	81.1	79.4	79.9	80.0
S. brunensis NRL/St 21/187	99.9	99.8	99.8	100	99.9	92.2	91.1	90.0	82.3	82.3	82.6	82.4	82.1	81.1	81.2	79.3	79.8	80.1
S. brunensis NRL/St 22/194	99.9	99.8	99.9	99.9	100	92.2	91.1	90.0	82.3	82.2	82.7	82.2	82.1	81.0	81.1	79.3	79.8	80.0
S. petrasii NCTC 13835^T	92.3	92.2	92.3	92.2	92.2	100	94.2	92.0	82.4	82.3	82.8	82.5	82.5	81.4	81.4	79.3	80.0	80.0
S. croceilyticus CCM 8421^T	91.1	91.1	91.1	91.1	91.1	94.2	100	92.1	82.0	82.4	82.7	82.3	82.2	81.3	81.2	79.4	79.8	79.7
S. pragensis CCM 8529^T	89.9	90.0	90.0	90.0	90.0	92.0	92.1	100	82.1	82.4	82.6	82.5	82.2	81.3	81.4	79.3	79.7	79.9
S. haemolyticus ATCC 29970^T	82.3	82.3	82.4	82.3	82.3	82.4	82.0	82.1	100	88.2	84.0	81.8	81.6	81.1	81.2	79.2	80.0	80.1
S. borealis strain 51-48^T	82.3	82.2	82.3	82.3	82.2	82.3	82.4	82.4	88.2	100.0	84.1	81.8	81.7	81.0	81.2	79.3	79.5	80.1
S. taiwanensis NTUH-S172^T	82.9	82.6	82.7	82.6	82.7	82.8	82.7	82.6	84.0	84.1	100	82.5	82.3	81.1	81.3	79.2	79.9	80.0
S. caledonicus H8-1^T	82.4	82.4	82.3	82.4	82.2	82.5	82.3	82.5	81.8	81.8	82.5	100	92.9	80.6	80.6	79.1	79.7	79.7
S. devriesi NCTC 13828^T	82.2	82.2	82.2	82.1	82.1	82.5	82.2	82.2	81.6	81.7	82.3	92.9	100	80.6	80.7	79.2	79.8	79.9
S. hominis subsp. *hominis* NCTC 11320^T	81.2	81.0	81.0	81.1	81.0	81.4	81.3	81.3	81.1	81.0	81.1	80.6	80.6	100	97.4	79.3	79.7	79.5
S. hominis subsp. *novobiosepticus* CCUG 42399^T	81.2	81.0	81.1	81.2	81.1	81.4	81.2	81.4	81.2	81.2	81.3	80.6	80.7	97.4	100	79.4	80.0	79.5
S. lugdunensis NCTC 12217^T	79.4	79.2	79.4	79.3	79.3	79.3	79.4	79.3	79.2	79.3	79.2	79.1	79.2	79.3	79.4	100	78.8	79.1
S. pasteuri DSM 10656^T	79.8	79.8	79.9	79.8	79.8	80.0	79.8	79.7	80.0	79.5	79.9	79.7	79.8	79.7	80.0	78.8	100	84.6
S. warneri NCTC 11044^T	79.9	80.0	80.0	80.1	80.0	80.0	79.7	79.9	80.1	80.1	80.0	79.7	79.9	79.5	79.5	79.1	84.6	100

Table S4. Variable phenotypic reactions of *Staphylococcus brunensis* sp. nov.

Strain No.	Phenotypic features				Antimicrobial susceptibility				Biolog Gen III															
	Catalase	Growth in 15% NaCl	Leucine arylamidase	α-Glucosidase	Ampicillin (2 µg)	Penicillin G (1 IU)	Erythromycin (15 µg)	Tigecycline (15 µg)	Dextrin	Sucrose	α-D-Lactose	N-acetyl β-D-mannosamine	D-Mannose	D-Arabitol	D-Fructose-6 PO4	L-aspartic acid	L-pyroglutamic acid	Pectin	Methyl pyruvate	L-Lactic acid	L-Malic acid	Tween 40	Acetoacetic acid	α-Keto butyric acid
NRL/St 16/872^T	+	+	+	-	S	S	S	S	b	b	+	-	-	-	b	+	+	b	+	+	b	+	+	-
NRL/St 18/288	+	+	-	-	R	R	S	S	+	+	+	+	+	+	b	+	+	+	+	+	+	-	+	+
NRL/St 19/737	-	+	+	-	R	R	R	R	-	-	-	-	-	-	-	b	+	+	+	+	+	+	+	-
NRL/St 21/187	+	-	-	-	S	S	S	S	-	-	-	-	-	w	-	b	b	-	-	-	-	b	b	-
NRL/St 22/194	+	+	-	+	S	S	R	S	b	b	-	-	-	b	+	-	-	b	w	-	-	-	-	-

+, positive; w, weak, b, borderline; -, negative; S, sensitive; R, resistant

Table S5. Summary of whole-genome sequence characteristics of analyzed *Staphylococcus brunensis* sp. nov. strains.

Strain	BioSample	GenBank Accession No.	Sequencing platform	Chromosome size (bp)	CG content (%)	CDS	rRNA	tRNA	Plasmid contigs (GenBank Accession No.)
NRL/St 16/872^{T}	SAMN25296428	CP119327.1	Illumina NextSeq, ONT	2,540,281	33.40	2472	19	62	CP119328.1, CP119329.1, CP119330.1, CP119331.1
NRL/St 19/737	SAMN25296429	JALGRI000000000.1	ONT	2,505,900	33.42	2558	19	61	JALGRI010000002.1, JALGRI010000003.1
NRL/St 18/288	SAMN25296430	JALGRH000000000.1	ONT	2,462,114	33.31	2662	19	61	JALGRH010000002.1, JALGRH010000003.1, JALGRH010000004.1, JALGRH010000005.1
NRL/St 21/187	SAMN25296431	JALGRG000000000.1	ONT	2,498,365	33.37	2554	19	61	JALGRG010000002.1, JALGRG010000003.1, JALGRG010000004.1, JALGRG010000005.1
NRL/St 22/194	SAMN32653323	CP116597.1	ONT	2,501,682	33.39	2702	19	61	CP116598.1, CP116599.1

Table S6. Pairwise nucleotide and amino acid identity matrices of chromosome cassette recombinases. See a separate spreadsheet file.

Table S7. Isolation sources of strains from the *Staphylococcus petrasii* complex identified in the National Reference Laboratory for Staphylococci, National Institute for Public Health, Prague.

Species	Total number of isolates	Ear infections	Wound infections	Bile	Blood sample	Urinary/sexual infections	Eye infection	Hospital enviroment	Other/unknown
S. brunensis sp. nov.	5	2	2	1	-	-	-	-	-
S. petrasii	214	17	55	1	96	18	5	5	17
S. croceilyticus	27	6	9	-	3	9	-	-	-
S. pragensis	133	9	34	2	27	53	2	-	6

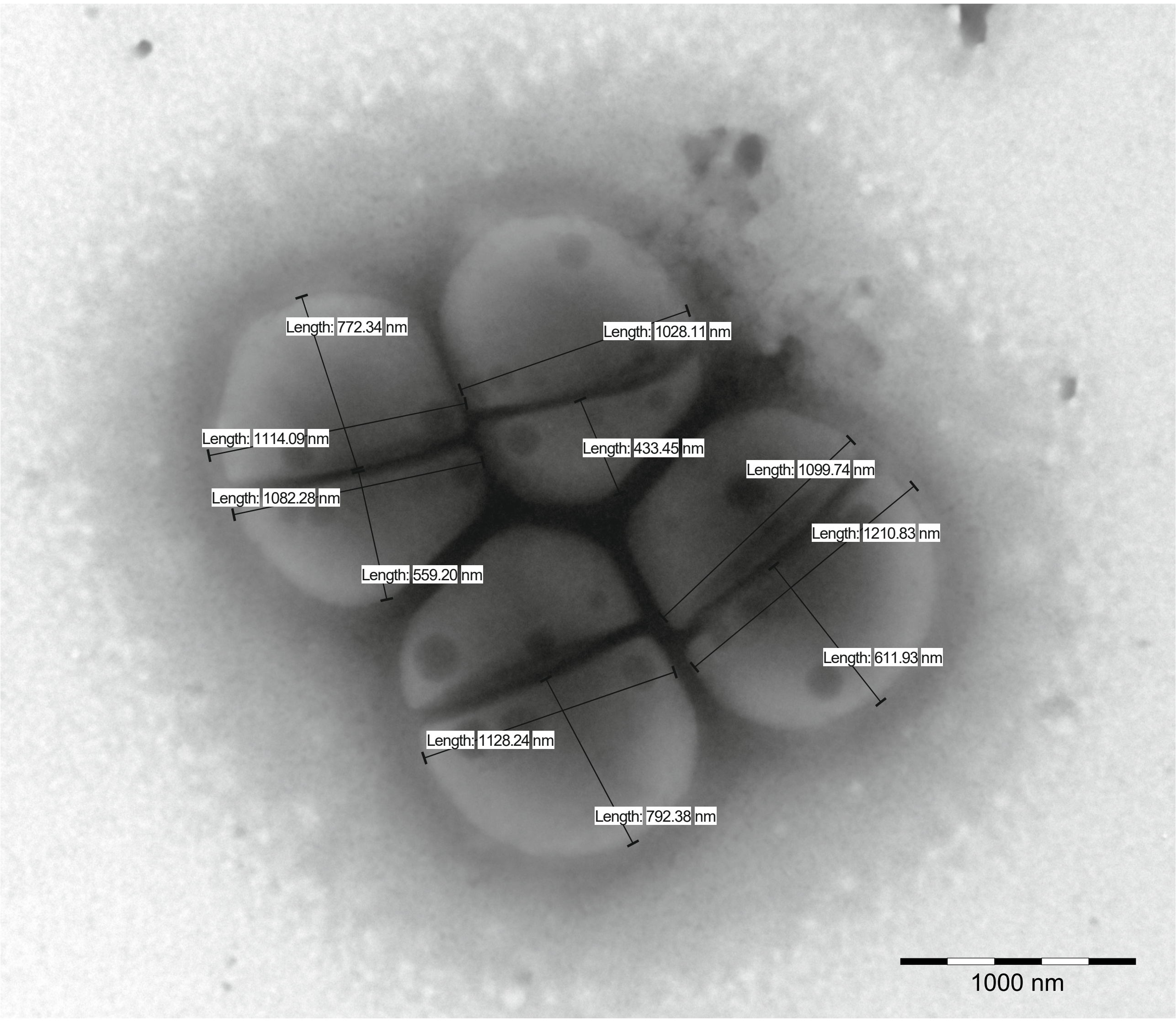

Figure S1. Transmission electron microscopy image of type strain *Staphylococcus brunensis* sp. nov. NRL/St 16/872^{T}, performed with Morgagni 268D Philips (FEI Company, USA) electron microscope. Negative staining with 2% ammonium molybdate. Bar represents 1,000 nm (original magnification, ×10,000).

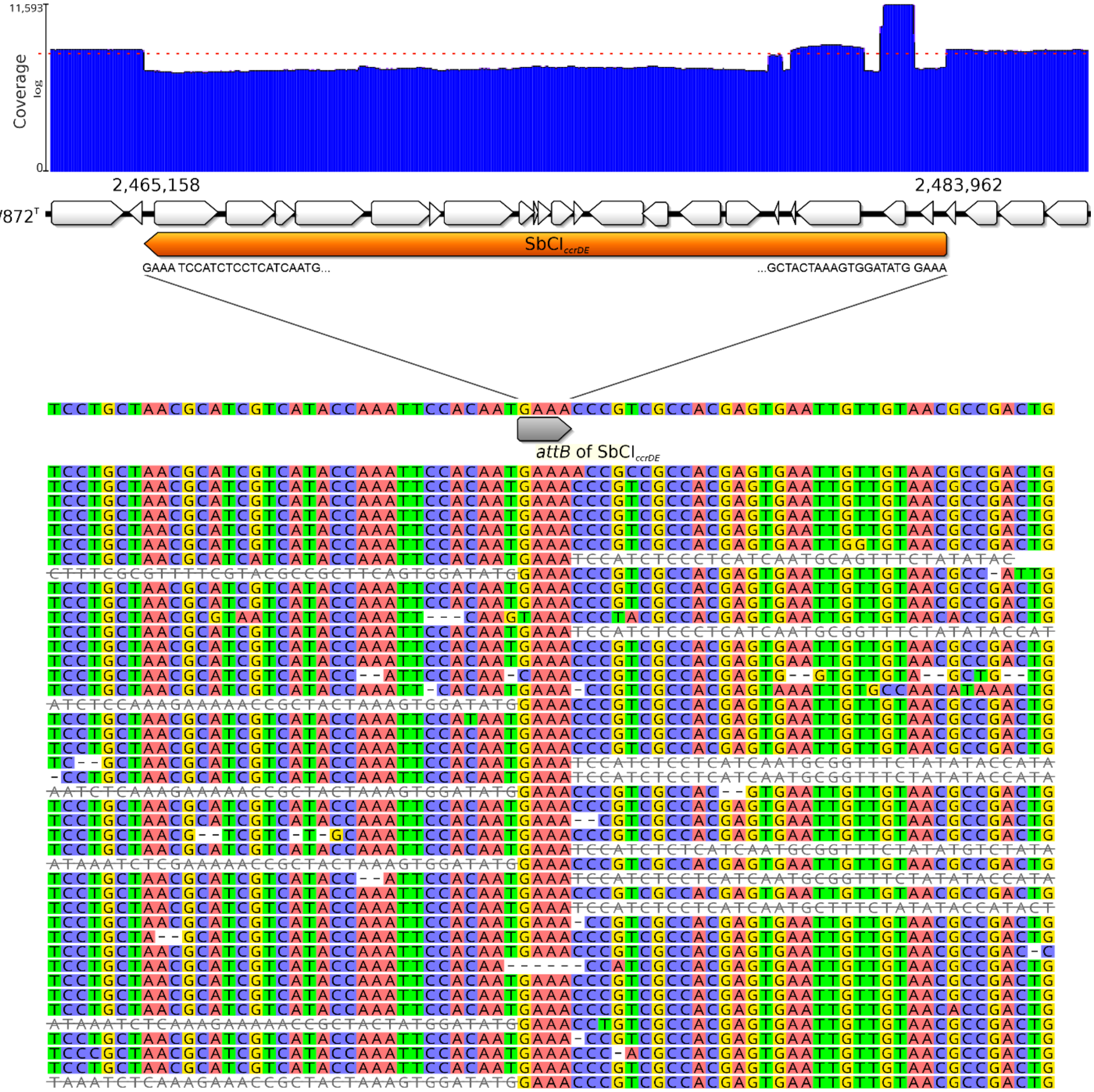

Figure S2. The sequencing coverage by ONT reads indicates the excision of SbCI$_{ccrDE}$ from the genome of *Staphylococcus brunensis* sp. nov. NRL/St 16/872^{T}. The mean coverage for the whole-genome sequence is indicated by red dashed line, the region containing SbCI$_{ccrDE}$ shows decreasing read coverage at the edges of the island. This decrease is due to the presence of SbCI$_{ccrDE}$-free genomes of strain NRL/St 16/872^{T}. The alignment of the long reads to the SbCI$_{ccrDE}$-free region is shown below. Reads that spanned a GAAA *attB* site and reads that mapped to the junction of the chromosome and SbCI$_{ccrDE}$ (shown in grey strikethrough text) can be observed.

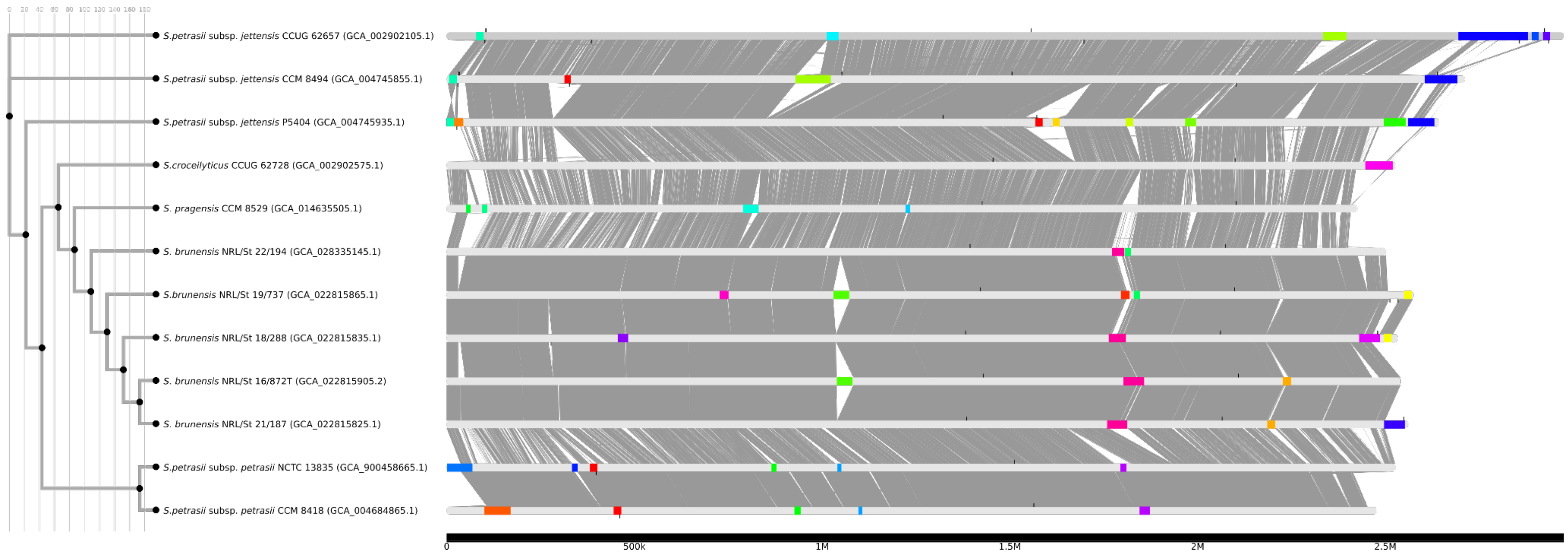

Figure S3. Comparison of variable genetic elements in the genomes of related strains belonging to the *S. petrasii* complex. The genomes of *S. brunensis*, *S. petrasii*, *S. pragensis*, and *S. croceilyticus* are aligned according to phylogenetic similarity based on the whole-genome sequences. Incomplete genome sequences were assembled based on the reference genome of *S. haemolyticus* JCSC1435 (GenBank accession number AP006716). Plasmid contigs are listed at the right end of the genome. Variable genetic elements are colour coded according to their similarity to each other. The analysis was performed in IslandCompare version 1.0.
